# Coded data quality for Casemix payment: insights from two external audits

**DOI:** 10.1186/1472-6963-11-S1-A8

**Published:** 2011-10-19

**Authors:** B Reid, A Coote, P O’Connor, J Berry, D Henry, C Aisbett

**Affiliations:** 1 Pavilion Health, Sydney, New South Wales, Australia; 2 Laeta, Sydney, New South Wales, 2031, Australia

## Introduction

Australia is currently undergoing a change in the Casemix payment environment. This is the result of an agreement to move to a more nationally-consistent approach to activity-based funding (ABF) for services provided in public hospitals. ABF for acute inpatients will be based on the Australian Refined DRG Casemix system, which is derived from the coded clinical data from each hospital admission. Thus, there will be a need to audit the clinical coding to assess the quality of the data in order to determine if the payments based on that coding are correct.

In 2009 and 2010, Pavilion Health conducted two major audits of clinical coding in NSW and Queensland. Together, these audits included 55 hospitals and 6,300 records. This paper discusses the insights gained from these two audits.

## Discussion

Errors in the coded data are not random. Some clinical conditions are more difficult to code than others, and some Major Diagnostic Categories (body systems) have more errors than others. Also, errors within hospitals are not random either. For example, one hospital had a relatively low predicted DRG mismatch of 5.6%, compared to 5.9% for the whole sample. However, it had a relatively large impact on case-weight change representing nearly A$8 million less in funding. One error in a high value DRG, repeated many times, was responsible.

The education and training of clinical coders varied. The resources needed by the clinical coding teams were not sufficient for the implementation of activity-based funding. In an ABF environment, additional tasks such as internal auditing, analysis, and consultation with clinicians require different and additional skill sets compared with the skills required to code competently.

## Conclusions

In order for Clinical Coders to meet submission deadlines and provide appropriate coding, they rely on comprehensive and timely clinical documentation. Clinicians and specialties have a responsibility to learn about coding and provide good documentation. There is a case for responsibility by Clinical Governance to audit clinical documentation for accuracy and completeness. Clinician engagement in and education on improving medical documentation is critical in order to improve the variation in Clinical Coding precision.

External coding audits are expensive to conduct and should be aligned with internal audits to gain the maximum educational value from the audits. Other tools such as error checking, both at the time of coding and later using the entire data set of the hospitals, also offer opportunities for improving coding accuracy beyond that afforded by external audits.

